# On Methods of Applying Silver Nitrate

**Published:** 1900-11

**Authors:** J. Morgan Howe


### Domestic Correspondence.

### ON METHODS OF APPLYING SILVER NITRATE.

To the Editor:

Sir,—In reply to Dr. Neeper's letter in the June number and to inquiries addressed to me, I would like to state in the Journal some of the methods that have been suggested and found useful of applying this silver salt to decaying tooth tissue.

Dr. Stebbins when he advocated the use of this agent suggested the use of a pointed stick, moistened in the saliva or in water, touched to the powdered salt, to carry the adhering particles to the wet cavity, and there rub them around gently till dissolved and brought into contact with the whole of the decayed surface. I think no more effective method than this has been suggested, but facility of application may be increased by using a piece of wire set in a handle instead of a stick, as the wire can be bent as required to make access easy to remote cavities. Some have found the use of a solution of the salt, in which pellets of cotton can be moistened, more convenient for general use, and I do not think any better way of keeping such solution has been suggested than that spoken of by Dr. McNaughton at the meeting of the New York Institute of Stomatology and published in the April number of the Journal, —namely, keeping a bottle nearly full of cotton saturated with a strong solution of silver nitrate, and then moistening pellets of cotton by pressing them against the saturated cotton in the vial.

The suggestion to fuse the crystals of silver nitrate on the end of a wire was originally made, I think, to facilitate treatment of pyorrhœa pockets with the salt. Quite a bulb of the fused salt can be made to adhere to the end of a wire by heating it and touching it repeatedly to small crystals and passing them through a flame to fuse them. This method of application affords facility of treating pockets of decayed surfaces, but it presupposes a surface covered with moisture to which it is to be applied in order that solution of the salt may take place on contact.

I have found it desirable to dry the parts outside of and around the place to be treated, in order that a flowing over of the caustic solution may not occur. I seldom dry cavities, but am careful to dry the mouth and soft tissues near the point of application; and after holding the mouth, so as to permit the action of the salt solution on the decayed surface, for fifteen to thirty seconds, I absorb the surplus solution from the cavity before directing the patient to rinse the mouth. This prevents the mouth and perhaps the fauces from being irritated.

I have not found the caustic action on gum tissue to be a serious matter. In treating cavities that extend to the gum, I wipe away the caustic solution from the gum as soon as the whitening effects of its contact is observed. Of course the gum could often be protected by using rubber dam, but without it I have not found the damage to the gum, resulting from the unavoidable action of silver nitrate in treating cervical cavities, to be nearly so damaging to that tissue as the preparation of cavities and introduction and finishing of fillings would generally effect.

The results of treating decayed tooth tissue with silver nitrate appear to be due entirely to the chemical transformation of the decalcified tissue into a new and stable compound. When the thickness of the strata of disintegrated dentine is considerable the action of the nitrate solution should be continued relatively longer in order to permit saturation of the deeper layers; otherwise renewal of the carious process is liable to occur under the superficial portion that has been rendered stable. I think that when the decalcified tissue is acted on, all the way through to the sound dentine under it, that the place so treated is always rendered immune for a long period, but care should be exercised to avoid such treatment of cavities of such depth as to permit a dangerous approach of the caustic to the pulp.

J. Morgan Howe.

